# Age-specific values of Access anti-Müllerian hormone immunoassay carried out on Japanese patients with infertility: a retrospective large-scale study

**DOI:** 10.1186/s12905-019-0752-z

**Published:** 2019-04-25

**Authors:** Tomoya Segawa, Kenji Omi, Yoshiaki Watanabe, Yaeko Sone, Masaki Handa, Masako Kuroda, Osamu Miyauchi, Hisao Osada, Shokichi Teramoto

**Affiliations:** 1 Shimbashi YUME Clinic, Excel Shimbashi, 2-5-1, Shimbashi, Minato-ku, Tokyo, 105-0004 Japan; 2 Natural ART Clinic Nihombashi, 8F Nihombashi Tower, 2-7-1 Nihombashi, Chuo-ku, Tokyo, 103-6028 Japan

**Keywords:** Anti-Müllerian hormone, Age-specific values, Japanese, Automated assay, Beckman coulter, Access immunoassay system

## Abstract

**Background:**

The ovarian reserve in women is known to correlate with anti-Müllerian hormone (AMH) levels, and currently the latest, third-generation, fully-automated AMH immunoassays, such as Access and Cobas, are beginning to be used for measuring AMH levels. However, the age-specific reference values obtained for AMH levels have been based on samples from an American population, measured using first-generation immunoassays. In this study, we attempted to determine the age-specific AMH reference values based on a large set of samples taken from Japanese infertile women measured by Access so that they could be used by infertility centers treating Japanese and those with similar racial and life-style characteristics.

**Methods:**

The study included 5483 Japanese patients who enrolled in infertility treatment programs at two in-vitro fertilization centers, Shimbashi YUME Clinic and Natural ART Clinic Nihombashi in Tokyo, and who had their serum AMH levels measured between December 2015 and November 2017 by Access. Each patient was represented only once in the study. The mean, median, and standard deviation values were obtained from the measured values for single-year intervals from 28 through 48 years of age (21 age groups in total). The 3D-fitted curve of age-specific mean and median values measured by Access was obtained by regression analysis.

**Results:**

The mean and median values decreased with advancing age (mean: R^2^ = 0.9864; median: R^2^ = 0.9926). In all age groups, the mean values were higher than the median values; however, the differences between these values decreased with increasing age.

**Conclusions:**

The age-specific AMH reference values measured by Access in this study may serve as a useful diagnostic marker in infertility centers, especially those treating Japanese patients or patients with similar characteristics.

## Background

The quantity and quality of a woman’s ovarian follicle pool are considered to be related to her age and follicle-stimulating hormone (FSH) level [1]. However, recent studies have shown that the number of follicles is well correlated with the level of anti-Müllerian hormone (AMH) [2], which is mainly produced by the granulosa cells of the preantral and small antral follicles in women [3, 4]. Serum AMH concentration correlates with the quantity of primordial follicles in the ovarian tissues [5], thus reflecting the number of dormant follicles in adult women. This is evidenced by the fact that AMH levels decrease with age [6–8]. Moreover, receiver operating characteristic curve analysis of serum AMH concentration has been reported to be a highly accurate diagnostic tool for polycystic ovary syndrome (PCOS) [9].

However, the reports regarding this relationship between AMH level and clinical results are contradictory. Some studies have reported that high AMH levels were associated with better clinical results [10, 11] whereas others reported the opposite [12–14]. The first generation of commercial AMH immunoassays was developed and marketed by Diagnostics Systems Laboratories, Inc. (DSL) and Immunotech (IOT), each using their own antibody. Beckman Coulter, Inc. then acquired both companies and developed a second-generation AMH immunoassay, namely the AMH Generation II (Gen II) assay, based on DSL’s antibody and IOT’s standards [4]. Gen II is based on an enzyme-linked immunosorbent assay.

Later, third-generation AMH immunoassays such as Access and Cobas were developed; these are based on the same antibody as Gen II and an automated measuring method using a chemiluminescent enzyme immunoassay, which has a higher sensitivity [15]. These fully automated AMH immunoassays offer many benefits, such as (i) elimination of manual operations, thus ensuring uniformity in data obtained from different institutions; (ii) provision for measurement of a single sample, thus eliminating the need for long-term sample preservation; and (iii) a reduction in measurement time, thus enabling outpatients to obtain AMH results during consultation on the same day. These third-generation AMH immunoassays have dramatically simplified the AMH measurement process in daily medical practice, and are now widely used in fertility centers.

When using an AMH marker in daily clinical practice, it is necessary to know its age-specific reference values. In 2011, Seifer et al. [6] reported the age-specific mean and median AMH values measured by DSL, the first-generation immunoassay, based on data from 17,120 infertile women visiting fertility centers in the United States of America. This study has been cited in many reports and the data are used as standard age-specific reference values of AMH in clinical practice. In our study, we attempted to evaluate retrospectively the age-specific AMH reference values based on a large set of samples taken from Japanese infertile women, measured by the fully automated Access immunoassay, and to derive the latest age-specific standard reference AMH values that can be used by infertility centers treating Japanese and those with similar racial and life-style characteristics.

## Methods

### Patients and AMH level measurements

Japanese women, aged between 28 and 48 years, who received infertility treatment at two in-vitro fertilization (IVF) centers (Shimbashi YUME Clinic and Natural Art Clinic Nihombashi) between December 2015 and November 2017 were included in the study. Serum AMH concentrations were measured by Access for all patients (*n* = 5483; age: 39.1 ± 4.0 years). In every patient, the AMH level was measured during the early follicular phase (between day 1 and day 5 of the menstrual cycle). As each patient was represented only once in the study, the first measurement was used for those who underwent multiple measurements.

### Data analysis

AMH data measured by Access were classified according to age, at single-year intervals from 28 through 48 years of age (21 age groups in total), and the age-specific mean, median, and standard deviation (SD) values were calculated. As the lower limit of detection for Access was 0.02 ng/mL, values lower than the detection limit were considered to be equal to zero. A regression analysis was performed for both the mean and median values, and the 3D-fitted curve was plotted to calculate the regression equation and the coefficient of determination (R^2^). The patients’ ages were divided into four groups and the incidence by the AMH value (integer part) was obtained. All analyses and the graph plotting were performed using JMP 11.2 (SAS Institute, Cary, NC, USA) and Microsoft Excel 2013 (Microsoft, USA).

## Results

The patient backgrounds were as follows: the BMI was 20.9 ± 2.7 kg/m^2^; the estradiol (E2), follicular stimulating hormone (FSH) and luteinizing hormone (LH) levels, which were measured at the same time, were 43.8 ± 21.9 pg/mL, 12.7 ± 8.3 IU/L, and 4.6 ± 2.6 IU/L, respectively. The smoking rate among the patients was 4.8%. The percentage of PCOS and POI (premature ovarian insufficiency) patients was 7.0 and 0.3%, respectively. The diagnosis of PCOS and POI was based on the literature from the Rotterdam ESHRE/ASRM-Sponsored PCOS Consensus Workshop Group [16] and Maclaran and Panay [17].

The ratios of the causes of infertility were 12.8, 42.3, 22.1, and 22.8% for male factor, female factor, both male and female factors, and unknown, respectively. There was no patient who used contraceptive pills in the previous cycle or any earlier cycles, as they were all visiting the clinics for their infertility treatment. We obtained the serum AMH concentrations of all of the patient groups, measured by Access. The mean, median, and SD values obtained for each age group are provided in Table 1.

**Table 1:** Mean, median, and standard deviation (SD) values obtained by Access anti-Müllerian hormone (AMH) immunoassays

Age (y)	n	Mean	Median	SD
28	30	5.4	4.5	4.3
29	54	4.6	4.4	2.7
30	71	5.3	3.9	4.6
31	125	4.5	3.7	3.6
32	187	4.7	3.8	4.8
33	201	4.3	3.5	3.3
34	254	3.7	3.0	2.9
35	313	3.4	2.8	2.8
36	375	3.4	2.8	3.0
37	416	3.0	2.4	2.8
38	498	2.7	1.9	2.5
39	542	2.4	1.8	2.1
40	485	1.9	1.4	1.8
41	554	1.6	1.2	1.4
42	478	1.5	1.0	1.5
43	384	1.3	0.9	1.4
44	236	1.1	0.8	1.2
45	132	0.8	0.4	0.9
46	73	0.6	0.5	0.7
47	46	0.6	0.5	0.6
48	29	0.6	0.2	1.1

(units: ng/mL)

**Fig. 1:**
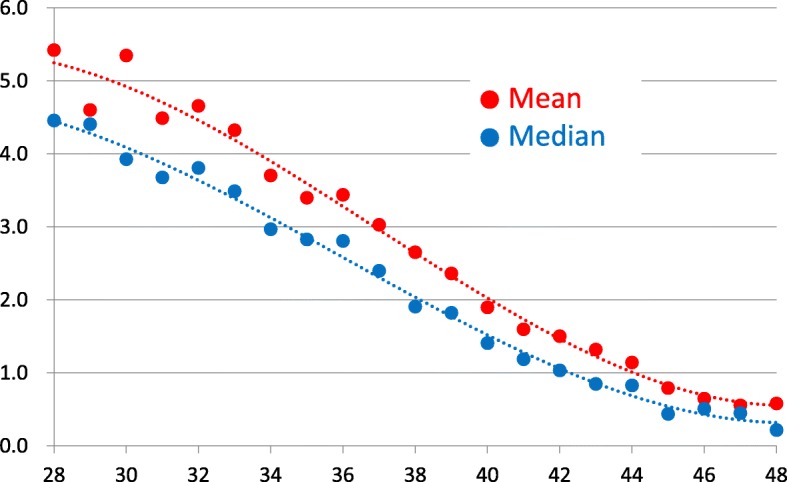
Age-specific median and mean anti-Müllerian hormone (AMH) values obtained by Access. Polynominal expression for mean values: y = 0.0008 × ^3^–0.0915 × ^2^ + 3.0608x – 26.84; R^2^ = 0.9864. Polynominal expression for median values: y = 0.0006 × ^3^–0.067 × ^2^ + 2.146x – 16.682; R^2^ = 0.9926. x-axis label: Age (years). y-axis label: AMH (ng/mL)

Figure 1 shows the 3D-fitted curve obtained by regression analysis of age-specific mean and median values measured by Access. The mean and median values decreased with advancing age (mean: R^2^ = 0.9864; median: R^2^ = 0.9926). In all age groups, the mean values were higher than the median values; however, the differences between these values decreased with increasing age.

**Fig. 2:**
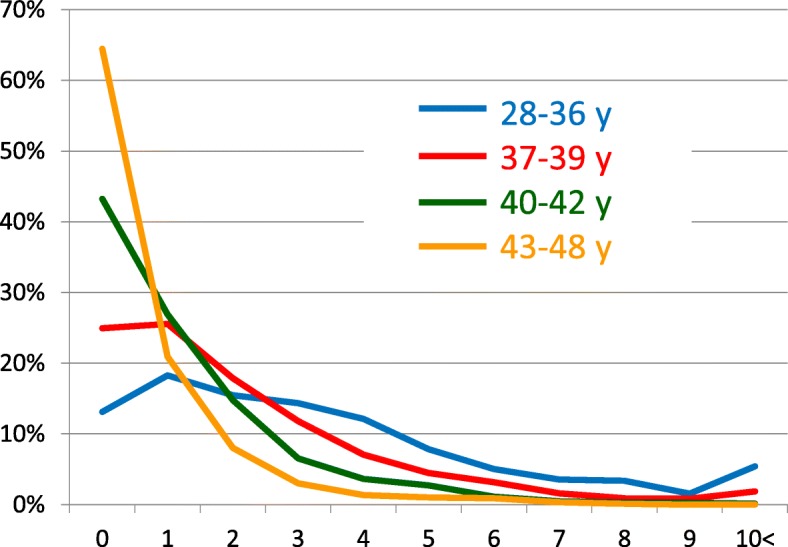
Distribution of anti-Müllerian hormone (AMH) values obtained by Access in 4 different age groups. x-axis label: AMH (ng/mL). y-axis label: Frequency of AMH value

Figure 2 shows the distribution of AMH values (calculated using the integer numbers only) within four different age groups (ages 28–36, 37–39, 40–42, and 43–48 years). All age groups showed skewed distributions of AMH values. Furthermore, higher age showed a skew towards lower AMH values.

## Discussion

The AMH immunoassay has been simplified for application in daily clinical practice, and it is widely performed during assisted reproduction and infertility treatment in Japan. However, age-specific reference values specific to Japanese patients derived from a large set of data have not been reported. Furthermore, the measurement methods have been repeatedly renewed in a relatively short period of time from the 1st- (DSL) to the 2nd- (Gen II), and then to the 3rd- (Access) generation immunoassays.

There have been some studies comparing the values obtained by DSL and Gen II but their observations were not consistent. Rustamov et al. [18] and Nelson et al. [19] reported that values measured by DSL were higher than those measured by Gen II, whereas Li et al. [20] found that values measured by Gen II were higher than those measured by DSL. Furthermore, Helden and Weiskirchen [15] reported that the values measured by Gen II were significantly higher than those measured by Access in women approaching menopause, but there was no difference in these values for women in other reproductive phases.

Pigny et al. [21] reported that the AMH values obtained by Access and Cobas, which is another automated AMH immunoassay distributed by Roche, were significantly lower than those generated by Gen II in non-PCOS, non-PCOM (polycystic ovary morphology) young women. In contrast, Pearson et al. [22], who divided the subjects into 5 age groups and compared Gen II and Access values at the 10th, 50th, and 90th percentiles, reported that both methods demonstrated a highly similar curvilinear relationship between AMH and age.

Some reports addressed these discrepancies and suggested that the variability of the data between institutions may be due to the difference in measuring methods. Bonifacio et al. demonstrated that the 2013 revision of the Gen II protocol of using 1:6 diluted serum samples produced higher values of AMH [23]. Craciunas et al. reported that AMH values decreased after 7 days when samples were left at room temperature [24].

The fully automated AMH immunoassays, such as Access and Cobas, are easier and more reliable for application in clinical practice than the 1st- and the 2nd-generation AMH immunoassays because they are less susceptible to operator error and the samples need not be preserved, as the results can be obtained immediately, thus providing uniform and stable results for all clinical practices.

Earlier, it was considered that the serum AMH concentrations do not vary significantly throughout the menstrual cycle [25]. However, recent studies have demonstrated variation of AMH levels in the follicular, ovulatory, and luteal phases [26, 27]. In this study, as all of the samples were collected during the early follicular phase, there was no possibility of variation due to the timing of sample collection. Some reports have also indicated that AMH values vary with race [27, 28], smoking, oral contraceptive use [7], and BMI [29, 30].

Age-specific AMH reference values for infertile women based on large samples of American women measured by DSL [6] and of German women measured by Access [31] have been reported. However, the rate of smoking and contraceptive pill usage in these populations is considered to be higher than that in the Japanese population. Although there are limitations in comparing the data obtained from different infertility centers and using different assays, the AMH values of Japanese women obtained through this study appear to be higher than those of Western women. Thus, this study that derived age-specific AMH reference values based on a large data set solely based on Japanese infertility patients may provide a useful marker for infertility treatment practices in Japan, which has a relatively high ratio of infertility patients.

This study is based solely on data from two centers in central Tokyo, which offer IVF treatment mainly based on natural or minimal ovary stimulation cycles, and all of the participants were Japanese. Furthermore, all of the laboratory technicians in both centers had been trained in the same laboratory and used the same protocol and techniques for measuring the serum AMH concentration. Therefore, the potential for data variability and bias based on patients’ racial or lifestyle characteristics or on the individual technician’s technique was minimal.

However, because values may vary according to the ethnicity of the patient, age-specific reference AMH values for Access in other ethnic groups may need to be ascertained through further study. We are also aware that the data in this study does not represent the age-specific AMH levels in the general population and that comparison to that needs further study.

## Conclusions

This study derived age-specific AMH reference values for the latest immunoassay in Japanese infertile women who have different characteristics from Western women. Henceforth, measurement of AMH levels will be a requisite test for IVF-based infertility treatment, particularly when selecting the ovary stimulation protocol and predicting the prognosis. The age-specific reference values provided in this study may serve as a useful diagnostic marker in infertility centers treating Japanese patients and patients racially similar to them.

## Abbreviations

AMH: Anti-Müllerian hormone
BMI: Body mass index
DSL: Diagnostics Systems Laboratory
FSH: Follicle-stimulating hormone
Gen II: Beckman Coulter AMH generation II
IOT: Immunotech
IVF: In-vitro fertilization
PCOM: Polycystic ovary morphology
PCOS: Polycystic ovary syndrome
POI: Premature ovarian insufficiency
SD: Standard deviation
